# A Thoughtful Act from Which Our Readers Derive Benefit

**Published:** 1913-03

**Authors:** 


**A Thoughtful Act From Which Our Readers Derive Benefit**

We guarantee an average of sixty reading pages a month, 720 a year, and, as will be noted, we are exceeding the guarantee. The standard of the JOURNAL has been increased from fifty-eight pages of reading and twenty-six of advertising to sixty of reading and thirty-six of advertising, in the last year and a half. This does not afford quite space enough for "reading notices" (which, you may have noted, are no longer inserted in the reading pages so-called, although they are worth reading), so that an extra form has to be added every few issues. Most of the extra form is used in the body of the JOURNAL. With this preamble, we will state that, in January one of our subscribers very kindly mentioned the JOURNAL as an advertising medium. A small, single-insertion advertisement was thus secured which paid for a couple of pages of good reading matter. Similar thoughtfulness will enable us to increase the size of the JOURNAL or to improve the quality, and, when the advertising section can be brought up to fifty pages, to reduce the subscription rate materially.

